# Physicochemical and Sensory Properties Colored Whey Protein-Cellulose Nanocrystal Edible Films after Freeze-Thaw Treatment

**DOI:** 10.3390/foods11233782

**Published:** 2022-11-23

**Authors:** Hongbo Sun, Xinnan Liu, Yue Huang, Xiaojing Leng

**Affiliations:** 1 Key Laboratory of Functional Dairy, College of Food Science and Nutritional Engineering, China Agricultural University, Beijing 100083, China; 2 Key Laboratory of Precision Nutrition and Food Quality, Ministry of Education, China Agricultural University, Beijing 100083, China

**Keywords:** whey protein isolate, cellulose nanocrystals, edible film, colorants, freeze-thaw, sensory properties

## Abstract

Balancing physicochemical properties and sensory properties is one of the key points in expanding edible packaging applications. The work consisted of two parts, one was to investigate the effects of cellulose nanocrystals (CNC) on the packaging-related properties of whey protein isolate films with natural colorants (curcumin, phycocyanin, and lycopene) under freeze-thaw (FT) conditions; the other was to test oral tactility and visual sensory properties of the edible films and their overall acceptability in packed ice cream. FT treatment reduced the mechanical strength and moisture content and increased the water vapor permeability of the films, as water-phase transformation not only disrupted hydrogen bonds but also the film network structure through physical stress. The oral tactility produced by CNC and the visual effect produced by colorants could affect participants’ preference for edible films. This study provides a good reference for the consumer-driven product development of packaged low-temperature products.

## 1. Introduction

Edible packaging materials can enter the mouth along with food and be safely consumed after oral processing through mastication, agglomeration, and lubrication via saliva [1]. Understanding the textural properties of edible packaging materials in oral processing is critical, as any edible material must satisfy consumer dietary preferences. Additionally, consumers expect packaged food to be visually appealing and provide a pleasant experience before consumption.

Colored edible packaging gives intuitive visual effects and can efficiently reduce the excessive addition of coloring agents to food itself, such as in individually packaged ice cream or ready-to-eat jelly. At present, after appropriate molecular modification or microencapsulation, some natural colorants, such as curcumin, lycopene, and phycocyanin, are gradually being introduced to dye edible packaging instead of synthetic colorants, to improve appearance, ensure food safety, and promote consumer acceptance [2,3,4]. Nevertheless, the sensory-related response to edible packaging is often largely underestimated, and an extensive number of studies are limited to optimized processing in packaging-related performance, which is misaligned with actual production needs. What consumers are looking for from real commercial edible films is an ideal sensual experience, as opposed to reliable packaging-related properties. Unfortunately, research on the sensory perception of edible films and its influences on product acceptability is lacking.

In edible materials, whey protein isolate (WPI) has become among the most suitable substrate for edible composite films due to its high nutritional quality, strong film-forming ability, and potential to effectively carry different functional components [5,6]. Considering a large number of foods need to be stored at low temperatures (chilled and frozen food), maintaining edible packaging performance is key in extending its applications. However, frozen products inevitably undergo freezing and thawing processes during transportation and reprocessing within the distribution chain and in domestic kitchens. After freeze-thaw (FT) processes, in contrast to traditional packaging materials, the physicochemical properties of WPI-based films may not meet packaging-related requirements. Local dehydration of proteins caused by moisture migration may damage the internal structure of films, leading to poor film performance [7].

Cellulose nanocrystals (CNC) from plant fibers are generally recognized as safe (GRAS) food additives and widely used in food products as a reinforcing agent, dietary fiber supplement, and low-calorie food filler [8,9]. Recent studies have demonstrated that CNC have the potential to relieve product performance changes caused by water-phase transformations [10,11,12,13]. Considering practical applications, the comprehensive improvement of the physicochemical properties and consumer sensory preference for colored protein packaging films is paramount under low temperature. To date, whether CNC addition affect WPI composite films containing colorants in terms of sensory properties and resistance to FT environments remains unknown.

Hence, the aim of this work was to search colored edible WPI-CNC packaging films suitable for packaging food in a low temperature environment and give rise to consumer preferences by investigating the effects of CNC on the physicochemical and sensory properties of films containing curcumin, phycocyanin, and lycopene under FT conditions. In particular, the oral and visual sensory performance of colored edible films in packed ice cream were emphatically investigated. This study is crucial in promoting the practical applications of edible packaging.

## 2. Materials and Methods

### 2.1. Materials

WPI powder with 90% protein content on a dry weight basis was purchased from Fonterra Co-operative Group (Auckland, New Zealand). CNC aqueous dispersion (8%, diameter: 4–10 nm, length: 100–500 nm, prepared by acid hydrolysis from wood pulp) was provided by Guilin Qihong Technology Co., Ltd. (Guangxi, China). CNC dispersion before use was ultrasonicated by an ultrasonic processor (NingBo Scientz Biotechnology Co. Ltd., Ningbo, China) with 300 W for 10 min to ensure good homogeneity. Food-grade NaOH was purchased from Angel Yeast corporation (Hubei, China). Food-grade HCl was purchased from Kunfeng Chemical Co., Ltd. (Jinan, China). Curcumin (90%) was purchased from Tekang Biotechnology Co., Ltd. (Zhengzhou, China). Water-soluble modified lycopene (10% lycopene, 40% OSA starch, and 10% maltodextrin as emulsifier) was obtained from Xihai Biotechnology Co., Ltd. (Xi’an, China). Phycocyanin (purity (A620 nm/A280 nm) >2.5) was obtained from Binmei Biotechnology Co., Ltd. (Taizhou, China). Distilled water was used for all sample preparations.

### 2.2. Film Preparation

Briefly, 5.0 g WPI and 2.5 g glycerol (as plasticizer) were dissolved in 50 mL of distilled water with continuous stirring for 2 h at room temperature as the stock solution. In addition, all sample preparations had the same stirring duration to prevent the influence of stirring parameters.

Since the addition of curcumin requires a specific alkali pretreatment process, to ensure comparability between films, except blank sample (WPI-1), all stock film-forming solutions were subjected to the same alkali treatment process so that the final pH values and ionic strengths remained constant.

#### 2.2.1. Native WPI (WPI-1) Film-Forming Solution

The WPI-1 film-forming solution was prepared as previously reported by Chai et al. [14], with some modifications. The protein was denatured by raising the pH of the stock solution to 8.0 with 3.0 M NaOH and heating the solution at 90 °C for 30 min, after which it was cooled to room temperature.

#### 2.2.2. WPI Alkalization-Neutralization (WPI-2) Film-Forming Solution

Preparation of the WPI-2 film-forming solution was performed as described by Taghavi Kevij et al. [4]. The pH of the reserve solution was raised to 12.0 with 3.0 M NaOH and stirred for 30 min in the dark. Subsequently, it was gradually reduced with 3.0 M HCl to pH 7.0.

#### 2.2.3. WPI and CNC Composite (WPI-3) Film-Forming Solution

After determining the appropriate CNC content (2.5, 5, 7.5, and 10% (*w/w*) of WPI dry weight) by preliminary experiments (data shown in Figure S1), CNC (5% (*w/w*)) were added to the WPI-2 film-forming solution, and the resulting WPI-3 film-forming solution was stirred continuously for 12 h.

#### 2.2.4. WPI-3 Film-Forming Solution Containing Colorants

WPI-3-phycocyanin (WPI-3-Phy) or WPI-3-modified lycopene (WPI-3-Lyc) film-forming solution: WPI-3 film-forming solution was added with phycocyanin (Phy; 0.2% (*w/w*) relative to WPI) or modified lycopene (Lyc; 0.2% (*w/w*) relative to WPI) under agitation in the dark for 1 h.

WPI-3-Cur film-forming solution: This solution was prepared according to the method proposed by Pan et al. [15], with modifications. The pH of the stock solution was increased to 12.0 with 3.0 M NaOH. Then, curcumin (Cur; 0.2% (*w/w*) relative to WPI) was added to the solution under agitation for 30 min in the dark, and the pH was gradually reduced to 7.0 with 3.0 M HCl; 5% CNC were added to this solution, and it was stirred in the dark for 12 h.

#### 2.2.5. Film Formation

After eliminating tiny particles and bubbles by ultrasonication, film-forming solutions were cast on Plexiglas plates (9-cm inner diameter) and dried at 40 °C for 24 h. Films were peeled off and equilibrated at 23 ± 2 °C and 50% ± 8% relative humidity (RH, prepared using magnesium nitrate saturated solution) for at least 72 h [4,14].

### 2.3. Freeze-Thaw Treatment

Films were subjected to FT seven cycles (each cycle: freezing for 24 h at −22 ± 3 °C (commercial refrigerator) and thawing for 1 h at 22 ± 3 °C in the dark). The preservation duration of non-freeze-thaw (non-FT) films and the films prepared under seven continuous freeze-thaw cycles (7-FT) was the same [16].

### 2.4. Film Characterization

#### 2.4.1. Thickness, Moisture Content (MC), Water Vapor Permeability (WVP), and Swelling Rate (SR)

Average film thickness (ten random sampling points, including center and perimeter) was determined for each type of film using a digital micrometer (Chengdu Chengliang Co., Ltd., Chengdu, China). The *MC* was determined by recording mass loss after oven-drying of small film strips (2-cm diameter) at 105 °C for 24 h and calculated as follows [17]:

(1) $$MC\left(\%\right)=\left(\frac{\Delta m}{m}\right)\times 100\;$$

where Δ*m* is the mass loss after drying and *m* is the initial mass. Five replicate measurements were performed for each film.

The WVP was determined by sealing cups containing calcium chloride (0% RH) with the films and placing the cups in a desiccator containing saturated NaCl solution (22 ± 1 °C, 75 ± 3% RH) [18]. The weight of the cups was recorded every 6 h during 48 h. The *WVP* was calculated as follows:

(2) $$WVP=\frac{\Delta m\times x}{A\times \Delta T\times \Delta p}$$

where Δ*m* is mass gain (g), x is film thickness (m), ΔT is exposure time (s), *A* is the area of the exposed film (m^2^), and Δp is the water vapor pressure differential across the film (Pa). Five replicate measurements were performed for each film.

Small film strips were pre-dried in an oven at 105 °C for 2 h to determine the initial dry weight ($m_{0}$); dried films were immersed in 50 mL of distilled water at 22 ± 3 °C for 5 min; then, films were blotted dry with tissue paper, and their weight recorded as $m_{1}$. *SR* values were calculated as follows [19]:

(3) $$SR\left(\%\right)=\left(\frac{m_{1}-m_{0}}{m_{0}}\right)\times 100\;$$

where $m_{0}$ is the initial dry weight and $m_{1}$ is the swollen weight. Five replicate measurements were performed for each film.

#### 2.4.2. Mechanical Properties

Tensile strength (*TS*) and elongation at break (*EAB*) of the composite films were measured using a texture analyzer (CT3, Brookfield Engineering Laboratories, Inc. Middleboro, MA, USA) according to Li et al. [20], with modifications. Ten-millimeter film samples were cut, at which a film area of 50 × 10 mm was left exposed after mounting the strip ends with double-sided adhesive tape between cardboard grips. The initial separation distance was set at 40 mm, and the film strips were stretched at a speed of 1 mm/s until the breakpoint. The *TS* (MPa) and *EAB* (%) were calculated as follows:

(4) TS=F/A

(5) $$EAB\left(\%\right)=\frac{\Delta L}{L_{0}}\times 100\;$$

where *F* is the maximum load (N) and *A* is the initial cross-sectional area of the specimen (mm^2^). Δ*L* is the change in initial length up to the breakpoint, and $L_{0}$ is the initial length. Six replicate measurements were performed for each sample.

Puncture strength (*PS*) and puncture deformation (PD) were measured according to Jiang et al. [17], after modifications. Film samples were placed over a centrally perforated polymethacrylate plate; the opening diameter of the hole was 3.2 cm, and the holder was fixed with four screws. Punctures were performed with a 2-cm cylindrical probe moving at 1 mm/s. PD (mm) was the fall distance of the probe before films were punctured, and *PS* (N/mm) was calculated as follows:

(6) PS=F/S

where *F* is the maximum puncture force (N) and *S* is the thickness of the film (mm). Six replicate measurements were performed for each sample.

#### 2.4.3. Attenuated Total Reflectance Fourier Transform Infrared (ATR-FTIR) Spectroscopy

Film samples were measured as described by Stoll, Rech, Flôres, Nachtigall, and de Oliveira Rios [3] using a Perkin Elmer Spectrum Two Infrared Spectrometer (Perkin Elmer, Hopkinton, MA, USA) in ATR mode with a wavenumber ranging from 4000 to 750 cm^−1^ at 32 scans with a resolution of 4 cm^−1^. In order to further determine changes in the protein secondary structure, the amide I region (1700–1600 cm^−1^) of the protein spectrum was curve-fitted using Peakfit software, v. 4.12 (SYSTAT Software, Richmond, CA, USA). For each region analyzed, a linear baseline was established, and the absorbance was normalized with respect to the peak maximum, to avoid undesirable intensity variations. To obtain reasonable fits, Fourier deconvolution was chosen to determine the values of the peak positions with several trials [21]. All the deconvolutions were carried out with R^2^ of 0.999.

#### 2.4.4. X-ray Diffraction (XRD)

Film samples were measured at room temperature using an X-ray diffractometer (Kristalloflex D500, Siemens, Mannheim, Germany) capable of monochromatic Cu-Kα radiation. The equipment was set at 40 kV and 40 mA. The scanning range of the diffraction angle (2 θ) was 4–48° with a step size of 0.02° and a scanning speed of 0.02°/0.1 s.

#### 2.4.5. Scanning Electron Microscopy (SEM)

The morphology of the cross-sections of the film samples was investigated via SEM (high-resolution, cold emission scanning electron microscope, Hituchi S-5500, Tokyo, Japan) at an accelerating voltage from 2 to 5 kV.

### 2.5. Sensory Evaluation

#### 2.5.1. Participants

The oral processing assessment was performed by a panel of 14 trained participants (seven males and seven females aged 24, on average) selected on strict dental criteria, who were graduate students majoring in food science. This study was approved by the Medical Ethical Committee of China Agricultural University (Project identification code: 20220204).

#### 2.5.2. Quantitative Descriptive Analysis

Participants received three training sessions (1 h/session) with the aid of sample standards to learn to identify and score sample-texture attributes (hardness, springiness, dissolution, cohesiveness, and adhesiveness, evaluation criteria are shown in Table S1), which they rated using a 9-point scale (1 being weakest and 9 being strongest), according to a previous study [22]. They then conducted evaluations of oral texture acceptability, appearance acceptability, and overall product acceptability (the product was presented as an example of packaged ice cream) using a 9-point hedonic scale (1 being “dislike extremely” and 9 being “like extremely”). Assessments were conducted at the Sensory Laboratory of the China Agriculture University under natural lighting.

#### 2.5.3. Procedure for Mastication Experiments

The participants were asked to chew each film sample (approximately 2.5 g) and to spit the boluses into corresponding containers just before deglutition, as described in a previous study by Lin et al. [23]. They were then asked to clean their mouth with water before measuring the next sample. The chew number (CN) and the chew duration (CD) were recorded.

#### 2.5.4. Product Eye Tracking Analysis

Six ice creams packed with different packaging films were displayed in an EyeSo Ec-80 eye tracker (60 Hz; Brain craft Technology Co., Ltd., Beijing, China) to further monitor the visual process of choosing each packed product by participants. The distance between the ET device (the 21″ Full HD screen resolution: 1920 × 1080) and the participants’ eyes was about 60 cm. At the formal measurement, participants were asked to follow the instructions: “Watch the image and use the mouse to select your favorite product.” The participants determined the duration of sample watching. ET metrics included total fixation duration and heatmap distribution [24]. The average fixation duration was the average of all participants’ fixation data on every product. The heatmap distribution was the visualized form of the total fixation duration data for all products, and the fixation duration was color-coded from long to short [25].

### 2.6. Statistical Analysis

Analysis of variance (ANOVA) was used to detect significant differences among films, and Bonferroni’s multiple comparisons test (*p* < 0.05) was conducted using GraphPad Prism 7.04 (GraphPad, Inc., San Diego, CA, USA). Spearman’s correlation analysis and principal component analysis (PCA) were used to understand film oral sensory characteristics, physicochemical properties, and the preference of the participants for these films. Spearman’s correlation coefficients were used to determine the relationships at a significance level of *p* < 0.05 using the SPSS software v. 22 (SPSS, Inc., Chicago, IL, USA). In PCA, varimax rotation on the correlation matrix using the SPSS software v. 22 and Kaiser criterion (eigenvalue > 1) was applied to identify the principal components.

## 3. Results and Discussion

### 3.1. Physicochemical Properties

#### 3.1.1. Mechanical Properties

**Figure 1:**
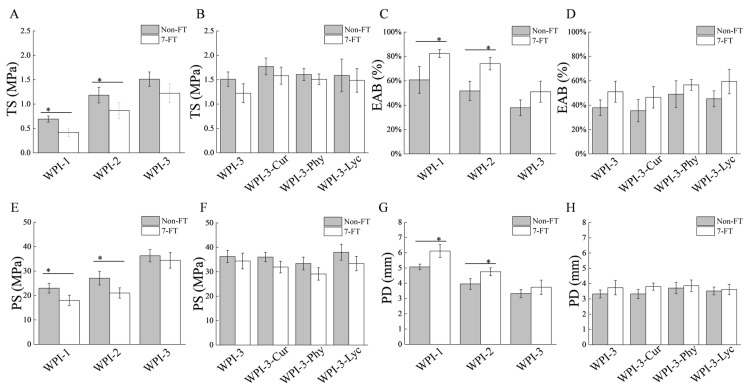
Comparison of the mechanical properties ((**A**,**B**) TS; (**C**,**D**) EAB; (**E**,**F**) PS; (**G**,**H**) PD) of films under non-freeze thaw (Non-FT) conditions and after seven continuous freeze-thaw cycles (7-FT). * Indicates significant difference at *p* < 0.05. TS (Tensile strength); EAB (elongation at break); PS (puncture strength); PD (puncture deformation); WPI-1 (native whey protein isolate film); WPI-2 (whey protein isolate alkalization-neutralization film); WPI-3 (whey protein isolate and cellulose nanocrystal composite film); WPI-3-Phy (WPI-3-phycocyanin film); WPI-3-Lyc (WPI-3-modified lycopene film); WPI-3-Cur (WPI-3-curcumin film).

Mechanical properties of the films under Non-FT and 7-FT conditions are shown in Figure 1. For films (Figure 1A,C,E,G) without FT treatment, the TS and PS of WPI-3 were higher than those of WPI-1 and WPI-2, while the EAB and PD of WPI-3 were lower than for WPI-1 and WPI-2. The degree of improvement was within a reasonable range compared with other literature [26,27]. This was attributed to hydrogen bonds between WPI and CNC, which enhanced the mechanical strength of the protein film network.

Furthermore, FT treatment reduced the TS and PS of WPI-1 and WPI-2 but increased the EAB and PD (*p* < 0.05). This was attributed to the moisture migration and the loss of hydrogen bonds between water and protein that caused localized matrix weakening to the protein film network structure, resulting in the decline of the rigidity of the films [28,29]. Notably, CNC addition effectively reduced changes in film mechanical strength under the FT treatment, likely due to the IRI activity of CNC as well as their action as physical barriers that effectively inhibited the migration of water molecules while maintaining the original film structure [12].

As for the WPI-3 film and its colored versions (Figure 1B,D,F,H), the addition of the different colorants did not significantly affect the mechanical properties of the films (*p* > 0.05), regardless of treatment. Indeed, colorants had the ability to decrease or interfere with the film network structure through H-bonding or other chemical bonds [2,4,30]. Therefore, the amount of colorants was controlled at a low enough level to avoid affecting the physical and chemical properties of the films but also providing the colorful appearance (cf. Section 3.2).

#### 3.1.2. Microstructure

**Figure 2:**
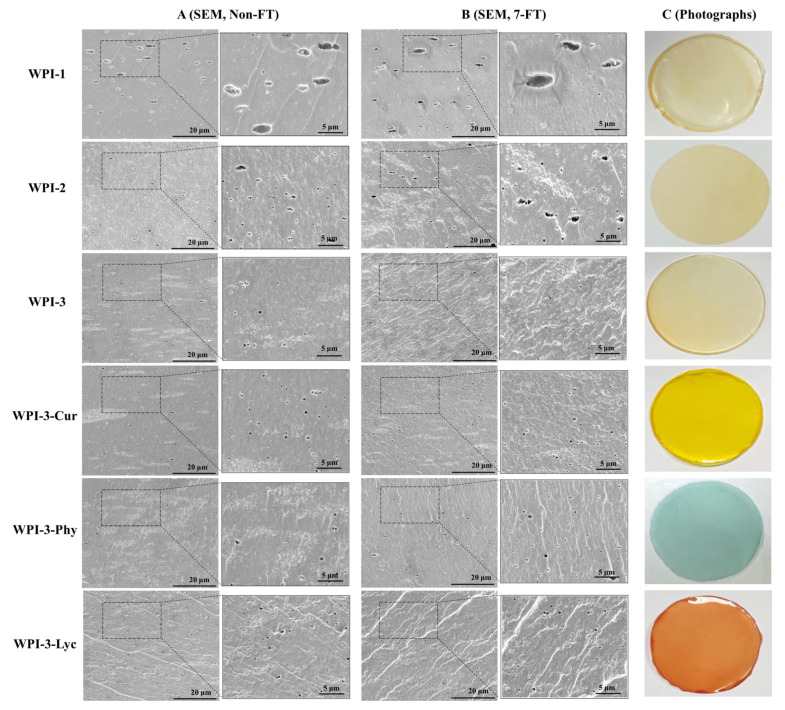
Comparison of scanning electron microscopy cross-sections ((**A**) non-FT treatment; (**B**) 7-FT treatment) of films and photographs without FT treatment (**C**). Scale bar = 20 μm. Non-FT (non-freeze thaw); 7-FT (seven continuous freeze-thaw cycles); WPI-1 (native whey protein isolate film); WPI-2 (whey protein isolate alkalization-neutralization film); WPI-3 (whey protein isolate and cellulose nanocrystal composite film); WPI-3-Phy (WPI-3-phycocyanin film); WPI-3-Lyc (WPI-3-modified lycopene film); WPI-3-Cur (WPI-3-curcumin film).

Figure 2A,B shows SEM cross-sections of films after non-FT and 7-FT treatments. Without FT treatment (Figure 2A), tiny pores appeared in WPI-1, which resulted from water evaporation and incomplete degassing during storage. The number of pores in WPI-2 decreased because the alkalization-neutralization process enhanced the interactions among molecules. Consistent with other reports, the number of pores was very small in WPI-3 because CNC addition enhanced molecular interactions within the film, thus compacting the film and reducing the availability of active sites for water adsorption [31]. After FT treatment (Figure 2B), the bigger pores appeared in WPI-1, compared with non-FT treatment, proof that the pure protein films were damaged by low temperature. Being strongly affected by temperature fluctuations, moisture migration and reduction of water-protein interaction changed the WPI-1 microstructure, resulting in larger pores due to localized matrix weakening and looseness of structure. Although alkalization strengthened the film structure, WPI-2 was still affected by the FT treatment. However, CNC addition maintained the average pore size in WPI-3 and its colored versions with FT condition. This result may have been caused by steric hindrance of the WPI-CNC network structure, which reduced diffusion kinetics and prevented moisture from migration and transformation, thus preserving the pore size [11].

#### 3.1.3. XRD Analysis

**Figure 3:**
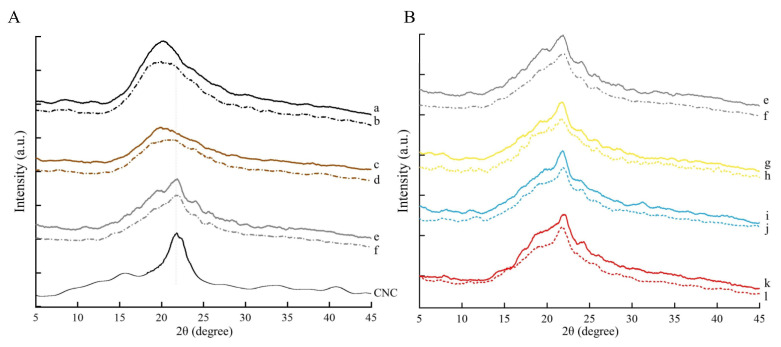
Comparison of X-ray diffractograms of films after non-FT and 7-FT treatments ((**A**) a,b: WPI-1; c,d: WPI-2; e,f: WPI-3; CNC. (**B**) e,f: WPI-3; g,h: WPI-3-Cur; i,j: WPI-3-Phy; k,l: WPI-3-Lyc). Solid and dashed lines indicate non-FT and 7-FT treatments, respectively. Non-FT (non-freeze thaw); 7-FT (seven continuous freeze-thaw cycles); cellulose nanocrystals (CNC); WPI-1 (native whey protein isolate film); WPI-2 (whey protein isolate alkalization-neutralization film); WPI-3 (whey protein isolate and cellulose nanocrystal composite film); WPI-3-Phy (WPI-3-phycocyanin film); WPI-3-Lyc (WPI-3-modified lycopene film); WPI-3-Cur (WPI-3-curcumin film).

X-Ray diffractograms of WPI-1, WPI-2, and WPI-3 films are compared in Figure 3A, and those of the colored WPI-3 films in Figure 3B, after non-FT and 7-FT treatments, respectively. Without FT treatment (Figure 3A), WPI-1 exhibited a broad peak at approximately 20°, which became wider and flatter in WPI-2, indicating that protein crystallinity decreased due to the alkalization-neutralization process [31]. A new peak in WPI-3 appeared at approximately 21.8°, contributed by CNC, as suggested by comparison with the diffractogram of pure CNC, and previously shown by Xiao, Liu, Kang, and Xu [30]. With FT treatment (Figure 3B), the peaks of WPI-1 and WPI-2 generally decreased and became flatter, which was caused by the low-temperature disruption of hydrogen bonds in the protein network structure, resulting in the destruction of the original crystalline domain in the protein network [28]. Although FT treatment reduced the crystallization peak of the WPI films, this effect was attenuated by the addition of CNC. This indicates that CNC inhibited disruption of protein conformation by inhibiting the migration of water molecules during FT processing. As expected, colorants addition had no effect on the films structure, regardless of treatment (Figure 3). Furthermore, there was good compatibility between the matrix and the colorants, indicating that the amounts of colorant used did not affect the crystallization characteristics of the films.

#### 3.1.4. ATR-FTIR Analysis

**Figure 4:**
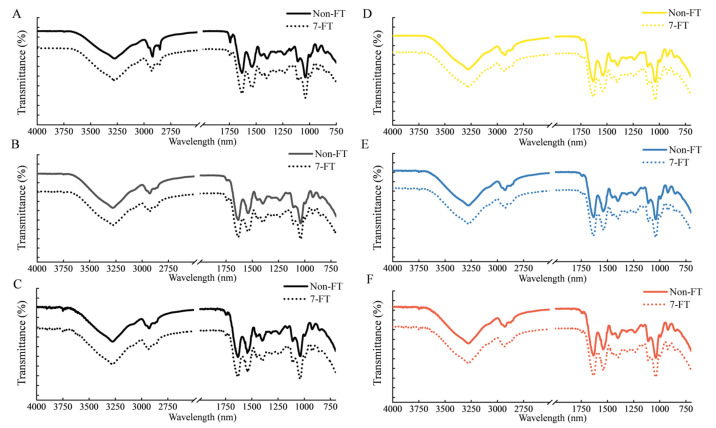
Comparison of the FTIR (Fourier transform infrared) spectra of the films. (**A**) WPI-1; (**B**) WPI-2; (**C**) WPI-3; (**D**) WPI-3-Cur; (**E**) WPI-3-Phy; (**F**) WPI-3-Lyc. Non-FT (non-freeze thaw); 7-FT (seven continuous freeze-thaw cycles); WPI-1 (native whey protein isolate film); WPI-2 (whey protein isolate alkalization-neutralization film); WPI-3 (whey protein isolate and cellulose nanocrystal composite film); WPI-3-Phy (WPI-3-phycocyanin film); WPI-3-Lyc (WPI-3-modified lycopene film); WPI-3-Cur (WPI-3-curcumin film).

**Figure 5:**
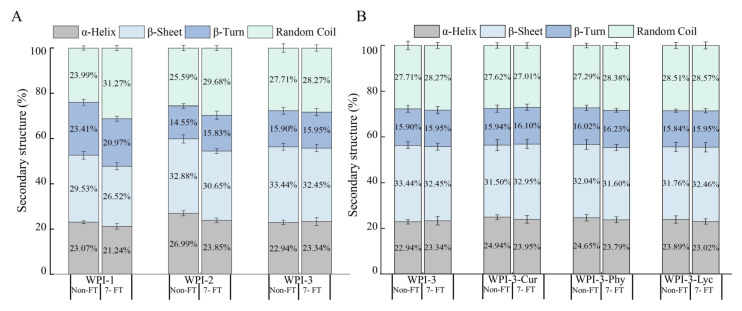
Comparison of the proportions of protein secondary structures in WPI-blended films (**A**), WPI-3 film and its colored versions (**B**), after non-FT or 7-FT treatment. Non-FT (non-freeze thaw); 7-FT (seven continuous freeze-thaw cycles); WPI-1 (native whey protein isolate film); WPI-2 (whey protein isolate alkalization-neutralization film); WPI-3 (whey protein isolate and cellulose nanocrystal composite film); WPI-3-Phy (WPI-3-phycocyanin film); WPI-3-Lyc (WPI-3-modified lycopene film); WPI-3-Cur (WPI-3-curcumin film).

The FTIR spectra of the WPI-blended films after non-FT and 7-FT treatments are shown in Figure 4. The characteristic peaks of WPI appeared in the regions of amide I (C=O stretching) at 1690 cm^−1^ and amide II (N-H deformation) at 1650 cm^−1^, respectively [32]. There were no significant differenes in FTIR spectra profile of all samples, regardless of treatment. It could be speculated that these treatments did not significantly change the chemical bonds and groups of the protein-blended films [33]. However, the protein secondary structure is generally sensitive to temperature fluctuations. Therefore, we compared changes in the protein secondary structure, including α-helix, β-sheet, β-turn, and random coil (Figure 5), obtained by spectral deconvolution.

With FT treatment (Figure 5), the random coil of WPI-1 and WPI-2 increased by 7.28% and 4.09%, respectively, and the conversion of α-helix and β-sheet to random coil indicated that moisture migration or water phase transformations resulted in the disruption of hydrogen bonds, which caused the protein secondary structure to become more unordered and protein molecules more flexible [34,35]. By contrast, the random coil of WPI-3 was less than 1%, indicating the effects seen for WP-1 and WPI-2 were attenuated by CNC [12,34]. In Figure 5B, the changes in the protein secondary structure of the remaining films were generally less than 2%, indicating that colorant effects were not important.

#### 3.1.5. Moisture Content, Water Vapor Permeability, and Swelling Ratio

The MC, WVP, and SR of the WPI-blended films after non-FT and 7-FT treatment are shown in Table 1. Without FT treatment, compared with those of WPI-1, the values of MC, WVP, and SR of the other films were significantly reduced (*p* < 0.05). After FT treatment, compared with non-FT treatment for WPI-1 and WPI-2, the WVP and SR of WPI-1 and WPI-2 were significantly increased (*p* < 0.05), and the MC of WPI-1 and WPI-2 were significantly decreased (*p* < 0.05). According to the work of Diefes et al. [36], Graiver et al. [37], and Pham and Mawson [38] moisture migration and water phase transformations caused loose and porous structures in the films, resulting in a decrease in water holding capacity and an increase in permeability and film expansion. In contrast, the MC and WVP of WPI-3 and its colored versions did not change significantly (*p* > 0.05), which was mainly attributed to the inhibitory effect of CNC on size and formation of ice recrystallization. However, the SR of WPI-3 changed significantly (*p* < 0.05), indicating that the IRI effect of CNC was limited and could not fully control the swelling of the film damaged by residual frozen water [39]. The properties of the films with colorants were basically the same as for those without colorants.

### 3.2. Oral Sensory Properties

#### 3.2.1. Correlation Analysis between Physicochemical Properties and Oral Sensory Parameters

**Figure 6:**
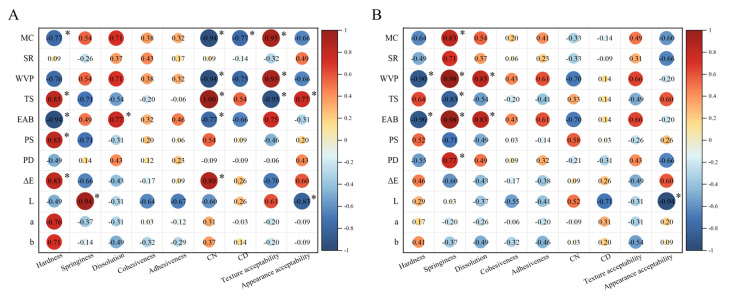
Spearman’s correlations between film physicochemistry and mechanic properties as well as sensory parameters ((**A**) non-FT treatment; (**B**) 7-FT treatment). * Indicates significant differences at *p* < 0.05. Non-FT (non-freeze thaw); 7-FT (seven continuous freeze-thaw cycles); MC (moisture content); WVP (water vapor permeability); SR (swelling rate); TS (tensile strength); EAB (elongation at break); PS (puncture strength); PD (puncture deformation); L (lightness); a (redness/greenness); b (yellowness/blueness); ΔE (total color difference); CN (chew number); CD (chew duration).

**Figure 7:**
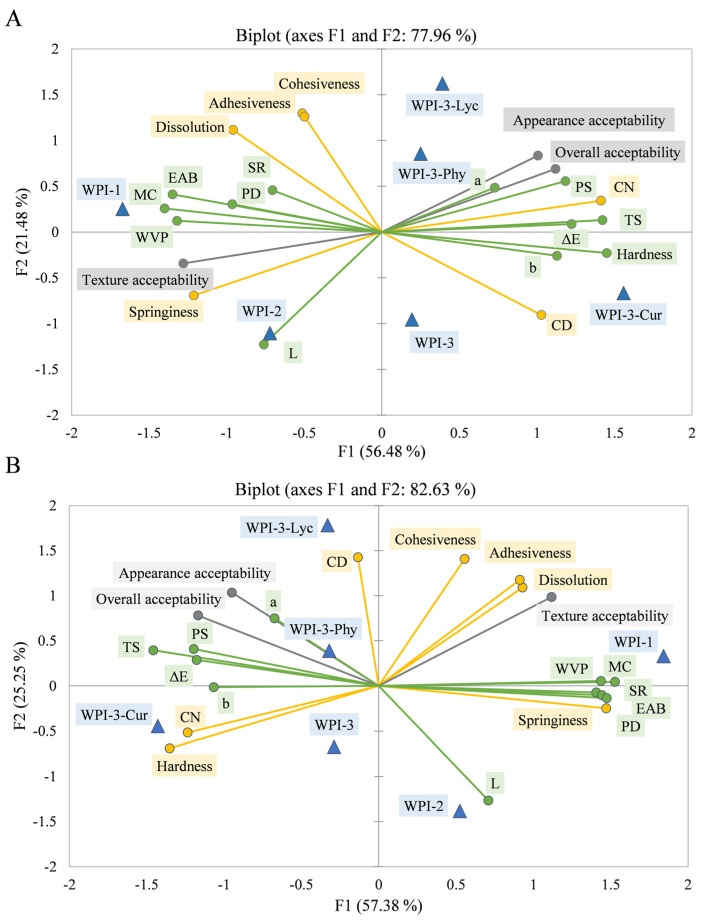
Bi-plot for principal component analysis of all films after non-FT (**A**) or 7-FT treatment (**B**). Blue triangles indicate film samples; green, yellow, and gray circles indicate physicochemistry and mechanic parameters, oral sensory parameters, and sensory preference, respectively. Non-FT (non-freeze thaw); 7-FT (seven continuous freeze-thaw cycles); WPI-1 (native whey protein isolate film); WPI-2 (whey protein isolate alkalization-neutralization film); WPI-3 (whey protein isolate and cellulose nanocrystal composite film); WPI-3-Phy (WPI-3-phycocyanin film); WPI-3-Lyc (WPI-3-modified lycopene film); WPI-3-Cur (WPI-3-curcumin film). MC (moisture content); WVP (water vapor permeability); SR (swelling rate); TS (tensile strength); EAB (elongation at break); PS (puncture strength); PD (puncture deformation); L (lightness), a (redness/greenness); b (yellowness/blueness); ΔE (total color difference); CN (chew number); CD (chew duration).

Spearman’s correlation coefficients of all films were compared after non-FT (Figure 6A) or 7-FT treatment (Figure 6B). Without FT treatment (Figure 6A), hardness strongly correlated with the MC, TS, EAB, and PS. Similarly, the CN significantly correlated with the MC, WVP, TS, and EAB. Furthermore, the CD and dissolution correlated significantly with the MC and EAB, respectively, indicating that the oral sensory perception of the participants was relatively sensitive to changes in the texture strength of the films, which suggested that the film strengthening effect of CNC was easily perceived. Notably, texture acceptability was strongly and positively correlated with the MC and WVP, and strongly and negatively correlated with the TS, i.e., the higher the strength and desiccation of the films, the lower the texture preference by the participants.

On the other hand, with FT treatment (Figure 6B), hardness strongly correlated with the WVP and EAB, while springiness strongly correlated with the MC, WVP, TS, EAB, and PD and dissolution significantly correlated with the EAB and WVP. These findings indicated that the decrease in film mechanical strength modified the participants’ feeling to springiness and dissolution. Additionally, the correlation between texture acceptability and other parameters was weak or moderate. Since color differences between non-FT and 7-FT films were difficult to distinguish, regardless of FT treatment, the appearance preference for both was strongly negatively correlated with lightness, not redness or blueness, parameters (Figure 2C shows photographs of the films, and Table S2 shows the color parameters).

#### 3.2.2. Principal Component Analysis

The first two principal components of the PCA for the WPI films after non-FT (Figure 7A) and 7-FT treatments (Figure 7B) explained 77.96% (Figure 7A) and 82.63% (Figure 7B) of the variance, respectively, which might reflect the main characteristics of the corresponding systems. In both PCAs, WPI-1, WPI-2, and WPI-3 were clearly divided into three different clusters, and WPI-3 and its colored versions were all on the same side of the ordination diagram. Furthermore, without FT treatment (Figure 7A), in terms of physicochemistry and mechanic parameters, along the F1 axis from left to right, the TS and PS of WPI-3 were higher than those of WPI-2 and WPI-1, while the SR, MC, PD, WVP, and EAB showed the opposite trend. As for oral sensory parameters, along the F1 axis from left to right, the CN and hardness of WPI-3 were higher than those of WPI-2 and WPI-1, while springiness showed the opposite trend. These observations are consistent with those reported by Foster et al. [40]. The differences between the WPI-3 film and its colored versions were mainly in oral sensory properties, mainly along the F2 axis. Similar results were observed for the FT treatment (Figure 7B).

**Figure 8:**
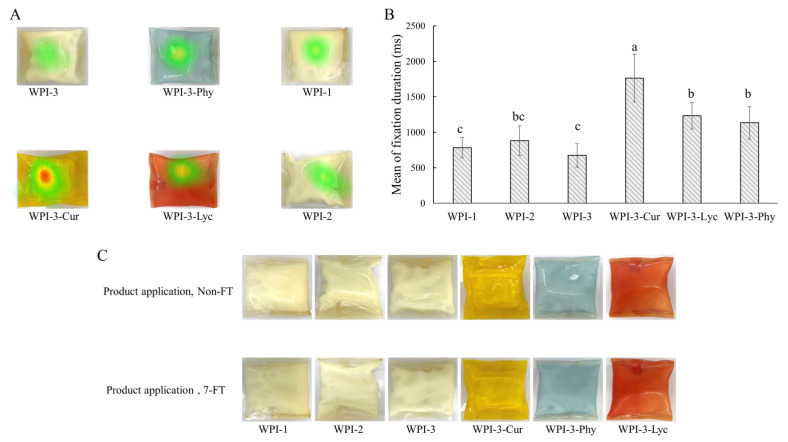
Heatmap images generated by the eye tracker (**A**). Average fixation duration (**B**); different letters indicate significant differences (*p* < 0.05). Product application images (packed ice cream with different edible packaging films) after non-FT or 7-FT treatment (**C**). Non-FT (non-freeze thaw); 7-FT (seven continuous freeze-thaw cycles); WPI-1 (native whey protein isolate film); WPI-2 (whey protein isolate alkalization-neutralization film); WPI-3 (whey protein isolate and cellulose nanocrystal composite film); WPI-3-Phy (WPI-3-phycocyanin film); WPI-3-Lyc (WPI-3-modified lycopene film); WPI-3-Cur (WPI-3-curcumin film).

After FT treatment, packaged ice creams with different films still had a good sealing and no difference in appearance compared with those of the non-FT treatment (Figure 8C). Texture acceptability was higher for WPI-1 and WPI-2 than for WPI-3 and its colored versions in both treatments, which suggested that the participants preferred edible films that were easier to chew. Appearance acceptability and overall product acceptability were both higher for colored films, indicating that a colorful appearance of products improved the overall product acceptability. Spence and Velasco [41] also came to a similar conclusion that packaging color might be the most important factor in multi-sensory product design.

#### 3.2.3. ET Analysis

To further understand the participants’ acceptance of each packed product, the visual distribution of the participants was analyzed by an ET system. Compared with native WPI packaging, the participants had a longer fixation duration and higher preference levels for colored packaging (Figure 8B). Correspondingly, the heatmap gave an intuitive visualization of the ET data (Figure 8A). Colored packaging attracted more attention, especially for yellow packaging containing curcumin, indicating that the areas of interest of the participants were mainly in colored packaging. These results revealed that the packaging color played a dominant role in enhancing product-standout and attracting the attention of consumers [41]. Therefore, to expand the practical applications in the design and development of edible packaging films, it is essential to use colorants to regulate appearance.

## 4. Conclusions

FT treatment reduced the mechanical strength and MC and increased the WVP and SR through physical damage to the film network structure. FTIR measurements showed that, due to the moisture migration, the hydrogen bonds were disrupted, causing the conversion of α-helix and β-sheet to random coil structures. XRD analysis indicated that FT treatment reduced the protein crystallinity of WPI-based films. CNC addition effectively attenuated these effects. Colorant effects on the physicochemical properties of the packaging films were minor due to the low amount used; however, future work should focus on these effects. Notably, although CNC addition improved the physicochemical properties of the films, it reduced their oral tactility preference. According to a combination of sensory data and ET analysis, incorporating colorants remedied this downside and achieved a high overall acceptance of the products. Hence, the key to the development of edible packaging applications in FT environments lies in obtaining a good FT stability and sensory acceptability.

## Figures and Tables

**Table 1:** Comparison of the moisture content (MC), water vapor permeability (WVP), and swelling rate (SR) of films.

	MC (%)		WVP (g × s^−1^ × m^−1^ × Pa^−1^)		SR (%)	
	Non-FT	7-FT	Non-FT	7-FT	Non-FT	7-FT
WPI-1	24.36 ± 1.04 ^a^	20.86 ± 1.02 ^a,^*	4.00 ± 0.25 ^a^	4.50 ± 0.11 ^a,^*	57.45 ± 4.48 ^a^	83.38 ± 5.29 ^a,^*
WPI-2	23.14 ± 1.32 ^a^	18.83 ± 1.15 ^b,^*	3.61 ± 0.16 ^b^	3.96 ± 0.18 ^b,^*	41.31 ± 4.01 ^b^	66.19 ± 3.23 ^b,^*
WPI-3	20.12 ± 1.13 ^b^	18.74 ± 1.14 ^b^	2.58 ± 0.15 ^c,d^	2.82 ± 0.24 ^c,d^	44.19 ± 4.71 ^b^	60.15 ± 3.62 ^c,^*
WPI-3-Cur	17.98 ± 1.20 ^c^	16.22 ± 0.83 ^c^	2.36 ± 0.29 ^d^	2.58 ± 0.27 ^d^	47.15 ± 2.94 ^b^	58.01 ± 3.28 ^c,^*
WPI-3-Phy	20.83 ± 1.41 ^b^	18.95 ± 1.13 ^b^	2.52 ± 0.32 ^c,d^	2.85 ± 0.33 ^c,d^	46.01 ± 4.33 ^b^	56.32 ± 4.13 ^c,^*
WPI-3-Lyc	20.75 ± 1.16 ^b^	19.24 ± 0.94 ^b^	2.85 ± 0.27 ^c^	3.09 ± 0.29 ^c^	46.15 ± 3.35 ^b^	59.35 ± 3.46 ^c,^*

Lowercase letter superscripts within the same column indicate significant (*p* < 0.05) differences among samples. * Indicates significant differences between treatments (*p* < 0.05). Non-FT (non-freeze thaw); 7-FT (seven continuous freeze-thaw cycles); WPI-1 (native whey protein isolate film); WPI-2 (whey protein isolate alkalization-neutralization film); WPI-3 (whey protein isolate and cellulose nanocrystal composite film); WPI-3-Phy (WPI-3-phycocyanin film); WPI-3-Lyc (WPI-3-modified lycopene film); WPI-3-Cur (WPI-3-curcumin film).

## Data Availability

All related data and methods are presented in this paper. Additional inquiries should be addressed to the corresponding author.

## Supplementary Materials

The following supporting information can be downloaded at: https://www.mdpi.com/article/10.3390/foods11233782/s1, Figure S1: Mechanical properties (A: Tensile strength; B: Puncture strength; C: Elongation at break; D: Puncture deformation) of WPI films with different CNC amounts (2.5–10%); Table S1: Attributes list and evaluation criteria; Table S2: Comparison of color parameters (Lab and ΔE) of films.
