# Supplementary material for: Physicochemical and Sensory Properties Colored Whey Protein-Cellulose Nanocrystal Edible Films after Freeze-Thaw Treatment
Source: Foods. 2022 Nov 23;11(23):3782. doi: 10.3390/foods11233782 (PMC9738979; doi:10.3390/foods11233782)
Supplement: Supplementary file 1 [file foods-11-03782-s001.zip › foods-2016690-supplementary.pdf]

Supplementary data

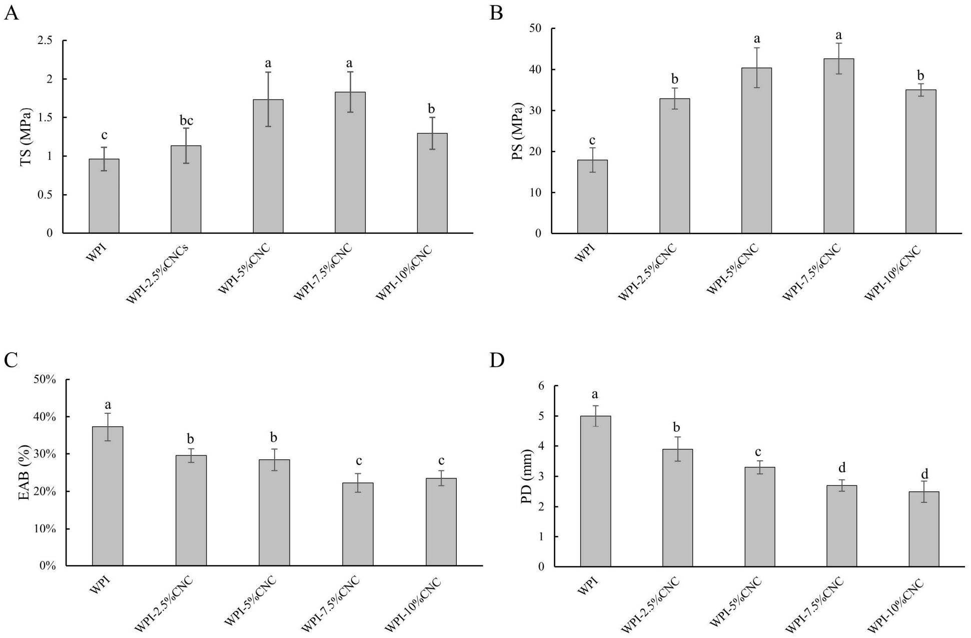

Figure S1. Mechanical properties (A: Tensile strength; B: Puncture strength; C: Elongation at break; D: Puncture deformation) of WPI films with different CNC amounts (2.5%-10%). Lowercase letters indicate significant difference at $P < 0.05$.

Figure S1 compares the mechanical properties of the WPI films with different amounts of CNC. With increasing of CNC concentration, TS and PS reached the maximum when the CNC concentration varied between 5-7.5%, while EAB and PD decreased monotonically. This indicated that an appropriate amount of CNC could effectively improve the strength of WPI film, but excessive amount could lead to a general decline in the mechanical properties of the film. This phenomenon was believed to be the destructive effect of CNC self-aggregation on the network structure of WPI film [1]. In view of this, the 5% CNC was thus chosen for the subsequent studies.

Table S1. Attributes list and evaluation criteria.

Descriptor	Definition	Reference
Hardness	The force to which cut completely through the sample when placed between incisive teeth.	Hard candy (9)
Springiness*	The degree to which the sample pieces return to their original size shape after partial compression between the tongue and palate.	Marshmallow (9)
Dissolution*	The degree to which sample pieces melt in the mouth.	Oblatum (9)
Cohesiveness*	The degree to which sample pieces hold together.	Chewing gum (9)
Adhesiveness*	The degree to which sample pieces stick to the palate or teeth (not sticky – very sticky).	Syrup (9)
Chew number (CN)	The number of which chewed each film sample (approximately 2.5 g) before deglutition.	/
Chew duration (CD)	The duration of which chewed each film sample (approximately 2.5 g) before deglutition.	/

*Springiness, dissolution, cohesiveness, and adhesiveness were evaluated after chewing 5-8 times.

Table S2. Comparison of color parameters (Lab and ΔE) of films.

	L		a		b		ΔE	
	Non-FT	7-FT	Non-FT	7-FT	Non-FT	7-FT	Non-FT	7-FT
WPI-1	85.34±0.36^{a}	85.17±0.37^{a}	0.11 ±0.14^{d}	0.25±0.08^{d}	8.30±0.63^{d}	9.46±0.73^{d}	11.70±1.64^{e}	11.21±0.36^{e}
WPI-2	85.84±0.19^{a}	85.57±0.19^{a}	1.58±0.37^{c}	1.77±0.48^{c}	15.26±0.62^{c}	14.52±0.88^{c}	16.78±0.37^{d}	16.31±0.49^{d}
WPI-3	85.49±0.27^{a}	85.64±0.81^{a}	1.65±0.18^{c}	1.61±0.36^{c}	15.02±0.37^{c}	13.25±1.64^{c}	16.77±0.68^{d}	15.21±1.27^{d}
WPI-3-Cur	77.50±0.71^{b}	78.06±0.26^{b}	16.19±0.54^{b}	16.4±0.27^{b}	85.43±0.81^{a}	86.07±0.18^{a}	87.67±1.54^{a}	87.58±1.11^{a}
WPI-3-Phy	74.36±0.58^{c}	72.82±1.04^{c}	-7.04±0.86^{e}	-6.91±0.09^{e}	5.54±0.89^{e}	6.38±0.47^{e}	21.84±0.86^{c}	23.40±1.18^{c}
WPI-3-Lyc	65.33±0.84^{d}	66.04±0.68^{d}	25.79±0.13^{a}	25.43±0.18^{a}	32.5±0.64^{b}	33.78±0.86^{b}	49.94±0.13^{b}	50.17±0.54^{b}

Lowercase letters indicate significant difference within the same column at $P < 0.05$.

References

1. Samadani, F.; Behzad, T.; Enayati, M.S. Facile strategy for improvement properties of whey protein isolate/walnut oil bio-packaging films: Using modified cellulose nanofibers. *Int. J. Biol. Macromol.* **2019**, *139*, 858-866, doi: https://doi.org/10.1016/j.ijbiomac.2019.08.042.
